# Coronary artery and thoracic aorta calcification is inversely related to coronary flow reserve as measured by ^82^Rb PET/CT in intermediate risk patients

**DOI:** 10.1007/s12350-013-9675-5

**Published:** 2013-03-07

**Authors:** Jongho Kim, Paco E. Bravo, Ali Gholamrezanezhad, Seil Sohn, Ash Rafique, Arlene Travis, Josef Machac

**Affiliations:** 1 Division of Nuclear Medicine, Department of Radiology, Mount Sinai School of Medicine, One Gustave L. Levy Place, Box 1141, New York, NY 10029-6574 USA; 2 Division of Nuclear Medicine, Department of Radiology, Johns Hopkins School of Medicine, 550 N. Broadway, Suite 300, Baltimore, MD 21205 USA; 3 Department of Neurosurgery, Seoul National University Hospital, Seoul, South Korea

**Keywords:** PET/CT, coronary flow reserve, coronary artery calcium, thoracic aorta calcium, coronary artery disease

## Abstract

**Background:**

The strength and nature of the relationship between myocardial perfusion imaging (MPI), coronary flow reserve (CFR), and coronary artery calcium (CAC) and thoracic aorta calcium (TAC) remain to be clarified.

**Methods:**

Dynamic rest-pharmacological stress ^82^Rb positron emission tomography/computed tomography MPI with CFR, CAC, and TAC was performed in 75 patients (59 ± 13 years; F/M = 38/37) with intermediate risk of coronary artery disease.

**Results:**

A total of 29 (39%) patients had ischemic and 46 (61%) had normal MPI. CAC was correlated with TAC (ρ = 0.7; *P* < .001), and CFR was inversely related with CAC and TAC (ρ = −0.6 and −0.5; *P* < .001, respectively). By gender-specific univariate analysis, age (*P* = .001), CAC (*P* = .004), and CFR (*P* = .008) in males, but CFR (*P* = .0001), age (*P* = .002), and TAC (*P* = .01) in females were significant predictors of ischemic MPI. By multiple regression, the most potent predictor was CFR [odds ratio (OR) = 0.17, *P* = .01), followed by age (OR = 1.07, *P* = .02), gender (OR = 4.01, *P* = .03), and CAC (OR = 1.002, *P* = .9).

**Conclusions:**

Combination of MPI, CFR, CAC, and TAC has complementary roles in intermediate risk patients.

**Electronic supplementary material:**

The online version of this article (doi:10.1007/s12350-013-9675-5) contains supplementary material, which is available to authorized users.

## Introduction

The strength and nature of the relationship between vascular calcification and its functional consequences on perfusion and vasoreactivity need to be clarified as a diagnostic as well as a prognostic biomarker. Vascular calcification is an anatomic change of atherosclerosis, occurring at highly variable rates in each gender, especially in the presence of plaques. In contrast, impaired vasodilator function reflects physiologic changes of vascular smooth muscle as well as endothelial function. Coronary artery calcium (CAC) and coronary flow reserve (CFR) represent different aspects of atherosclerosis, highlighting that, although their mutual correlation is weak, both of them may have their respective important roles.^1^

CAC has substantial incremental prognostic value beyond clinical risk factors (RF) among asymptomatic adults.^2^,^3^ Similarly, the additional prognostic value of CFR beyond clinical RF and myocardial perfusion imaging (MPI) has been established in symptomatic patients.^4^ In contrast, although CAC may have incremental prognostic value beyond MPI,^5^ even among patients with low or zero CAC a significant proportion of symptomatic patients may have obstructive coronary artery disease (CAD) on angiography.^6^-^9^ As a result, symptomatic individuals may be more likely to benefit from CFR assessment rather than CAC.^1^

Thoracic aorta calcium (TAC) has been proposed as a probable independent predictor of cardiovascular disease (CVD)^10^-^16^ as comparable as CAC especially for pre- and peri-menopausal women because of a higher prevalence and magnitude of TAC compared with lower CAC. Therefore, the aim of the present study was to assess the relationship between MPI, CFR, and CAC as well as TAC obtained from rest and pharmacologic stress ^82^Rb positron emission tomography (PET)/computed tomography (CT) in patients with intermediate risk for CAD.

## Methods

### Subjects

Seventy-five consecutive patients with intermediate likelihood of CAD who completed combined rest-stress ^82^Rb PET/CT MPI without evidence of transmural myocardial infarction at Mount Sinai Hospital, NY, were retrospectively analyzed. All patients were referred for pharmacologic (dipyridamole or adenosine) stress PET/CT MPI on clinical grounds. (See Supplement) Global and regional CFR, CAC, and TAC scorings were measured.

### Cardiac ^82^Rb PET/CT MPI

As a routine preparation for ^82^Rb cardiac PET/CT, patients were asked to discontinue taking nitrates for 6 hours, calcium-channel blockers and caffeine-containing beverages for 24 hours, and β-blockers for 48 hours before their appointment. Scans were acquired using a GE Discovery DLS BGO PET/16-Slice CT scanner (GE, Milwaukee Wisconsin, USA). Rest and stress cardiac PET/CT images were acquired as follows: For the rest images, a scout CT was performed to check the patient position. Subsequently, a slow CT attenuation correction scan was performed (140 kV, 40 mAs, 16 × 0.625 mm collimation, 5 mm slice), covering the thorax in 16s under shallow breathing. Then, from 1,480 to 2,220 MBq (40-60 mCi) of ^82^Rb were injected intravenously, and a 7-minute dynamic PET study was acquired; the acquisition was started at the very beginning of the ^82^Rb injection. Pharmacologic stress was then administered using adenosine (0.14 mg/kg/minute for 4 minutes) or dipyridamole (0.142 mg/kg/minute for 6 minutes). A second dose ranging from 1,480 to 2,220 MBq (40-60 mCi) ^82^Rb was injected at peak hyperemia, and a 7-minute dynamic PET study was acquired, in 30-second frames.^17^ The dynamic PET MPI images were reconstructed by filtered back-projection, after correction for dead-time, scatter, and random coincidences. Images were reconstructed using filtered back projection with Hanning filter. The qualitative results of MPI studies were semi-quantitatively interpreted by an experienced nuclear cardiologist based on a 17-segment American Heart Association scoring system^18^ from the rest and stress images produced from the dynamic MPI images summed between 2 and 7 minutes. The images were classified as normal or ischemic MPI, which were further classified as having mild, moderate, or severe ischemic disease, based on the most abnormal regions.

### Coronary Artery and TAC Scoring

Before MPI, the patients underwent CT for CAC and TAC scoring on an integrated 16-slice multidetector CT scanner (collimation, 4 × 2.5 mm; gantry rotation time, 500 ms) using prospective gating at 60% of R-R interval. This gated CT scan (120 kV, 400 mA) was acquired and reconstructed with filtered back-projection and a standard convolution kernel to 2.5-mm slices with a 512 × 512 matrix and a fixed 25-cm field of view. CAC and TAC scores and volumes were determined on an external workstation commercially available (Advantage Windows, version 4.4.1 and 4.2, General Electric Medical Systems) using CAC-scoring software (version 3.5, Smartscore). The global CAC and TAC scores were further classified into three categories: 0 (calcium absent), 1 to 400, and ≥400 for CAC; and 0 (calcium absent), 1 to 1000, and ≥1000 for TAC (See Supplementary Material).

For the measurement of TAC scores, three regions of thoracic aorta were analyzed: the ascending aorta, aortic arch, and the descending thoracic aorta. The arch comprised the origin of the aortic arch (defined as the image in which the ascending and descending aorta merge into the inner curvature of the aortic arch) to the first 1 cm of the origin of left subclavian and common carotid arteries and the brachiocephalic artery. The ascending aorta and the descending thoracic aorta were identified proximal, and distal to the aortic arch, respectively.

### Quantification of CFR

Myocardial perfusion at rest and during stress was calculated from the global and regional myocardial uptake divided by the summed blood pool activity and by the extraction fraction of ^82^Rb, the latter being based on a measured relationship between perfusion and the extraction fraction in dogs, as described by Yoshida et al,^19^ for their simple model. The dynamic images were summed between 3 and 5 minutes after the start of ^82^Rb infusion to produce the myocardial uptake images. The left ventricular myocardial activity was determined from the short-axis images and displayed as a polar plot with 33 sectors using an automated program. The dynamic images were summed between 0 and 5 minutes to produce the summed blood pool images. The blood pool activity was determined manually from a region of interest placed in the middle of the left atrium on vertical long-axis images. Corrections for the partial volume effect and tissue cross-talk were applied. The CFR was defined as the ratio between stress and resting myocardial blood flow. The normal CFR cut-off value was 2.0^20^ (See Supplementary Material).

### Statistical Analysis

Data are expressed as mean ± SD, and ordinal variables are summarized by count and percentages. The intra-observer variability for CAC scoring, TAC scoring, and CFR measurement were evaluated by the intraclass correlation coefficient (ICC). Receiver operating characteristics (ROC) analysis was used to compare the use of CAC, TAC, and CFR, for predicting ischemic PET-MPI. Independent two sample-test for the variables with asymptotic normal distribution or nonparametric Mann-Whitney U test for the variables were used. Logistic regression analysis with both single and multiple predictors including interaction variables of gender was performed to evaluate the dependency/non-dependency of the parameters on ischemic MPI to determine the predictors. In order to find the best predictors for logistic model, both forward and backward stepwise methods with likelihood ratio statistic were applied where candidate variables with a *P* value <.05 were entered, and those with a *P* value >.10 were removed from the model. Data analyses were performed using SPSS 18 or Intercooled Stata 9.2 Windows^®^ version. In all assessments, *P* < .05 was considered significant. Bonferroni corrected significance levels were also expressed for adjusting familywise error rate (See Supplementary Material).

## Results

Pertinent clinical characteristics of the patient population, stratified by PET-MPI result, are shown in Table 1. Out of 75 patients, 29 (39%) had ischemia, of which 17 had mild, five moderate, and seven severe ischemia. Forty six (61%) patients had normal MPI. Cardiovascular RF were comparable between patients with normal and ischemic MPI-PET. However, patients with ischemic MPI were significantly older, more likely to be male, had higher mean CAC and TAC scores and lower CFR values and LVEF as compared to patients with normal PET-MPI (Table 1). Intra-observer variability for measurements for CAC, TAC, and CFR in 50 randomly selected patients were 11%, 5%, and 4%, respectively (ICC > 0.9; *P* < .001 in all three markers). Despite significant intraobserver variability for CAC compared with CFR and TAC, respectively, the percentile ranking assigned to the two observes differed in only 8% (4/50) of patients.

**Table 1:** Baseline characteristics of patients

Parameter	Overall (n = 75)	Normal PET MPI (n = 46)	Abnormal PET MPI (n = 29)	*P* value
Age	59 ± 13	54 ± 1	67 ± 12	.00001
Male gender	37 (49%)	17 (37%)	20 (69%)	.009
Diabetes	21 (28%)	11 (24%)	10 (35%)	ns
Hypertension	47 (63%)	27 (59%)	20 (69%)	ns
Hyperlipidemia	42 (56%)	24 (52%)	18 (62%)	ns
Smoking	7 (9%)	4 (9%)	3 (10%)	ns
Family history	16 (21%)	12 (26%)	4 (14%)	ns
BMI	32 ± 10	32 ± 9	32 ± 10	ns
CAC score	288 ± 647	59 ± 182	651 ± 911	.0001
CAC percentile	55 ± 31	46 ± 32	69 ± 25	.001
CFR	2.1 ± 0.5	2.3 ± 0.5	1.8 ± 0.5	.00001
TAC score	877 ± 2,244	445 ± 1,562	1,563 ± 2,932	.03
Rest				
HR	71 ± 11	70 ± 10	71 ± 13	ns
SBP	134 ± 24	131 ± 26	139 ± 20	ns
DBP	76 ± 14	76 ± 15	77 ± 11	ns
RPP	9,523 ± 2,550	9,272 ± 2,419	9,921 ± 2,741	ns
Stress				
HR	92 ± 14	94 ± 13	89 ± 15	ns
SBP	125 ± 19	123 ± 21	128 ± 17	ns
DBP	68 ± 12	69 ± 14	68 ± 10	ns
RPP	11,551 ± 2,644	11,587 ± 2,662	11,493 ± 2,662	ns
% d RPP	0.2 ± 0.2	0.3 ± 0.2	0.2 ± 0.2	ns
SRS	1.2 ± 4.4	0.3 ± 0.8	2.6 ± 6.8	.03
SSS	3.3 ± 6.5	0.4 ± 0.9	7.9 ± 8.6	.00001
SDS	2.1 ± 4.5	0.1 ± 0.4	5.4 ± 6.0	.00001
LVEF	55 ± 12	57 ± 9	52 ± 11	.05

Values are mean ± SD, n (%), Bonferroni corrected *P* < .005.

*PET MPI*, Positron emission tomography myocardial perfusion image; *BMI*, body mass index; *CAC*, coronary artery calcium; *CFR*, coronary flow reserve; *TAC*, thoracic aorta calcium; *HR*, heart rate; *SBP*, systolic blood pressure; *DBP*, diastolic blood pressure; *RPP*, rate pressure product; *% d RPP*, % difference rate pressure product; *SRS*, summed rest score; *SSS*, summed stress score; *SDS*, summed difference score.

### The Relationships of CAC, TAC, and CFR with Severity of Ischemia

**Figure 1:**
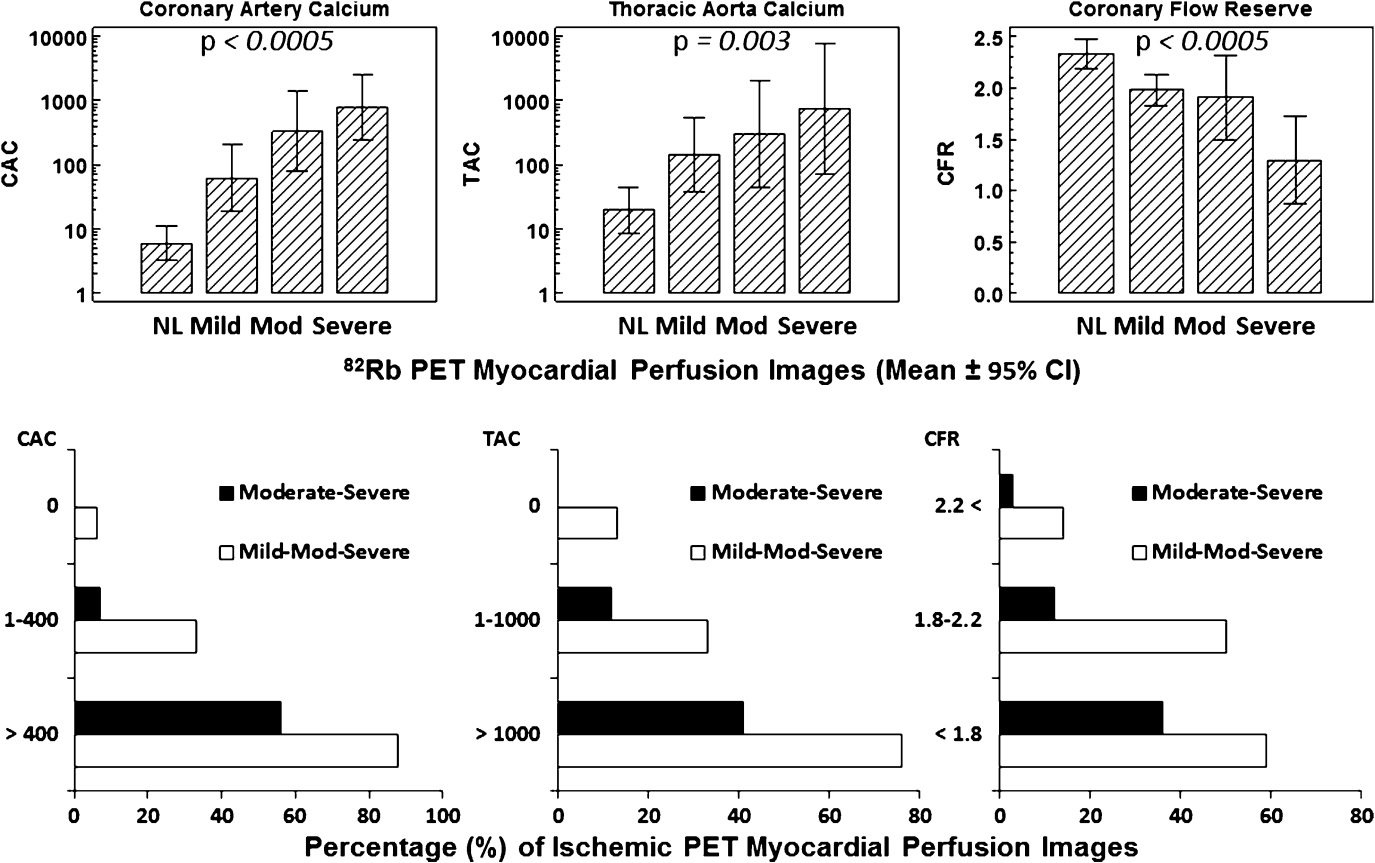
CAC significantly increased with the severity of ischemic MPI (*upper left*); and the proportions of both mild-to-severe ischemic MPI and moderate-to-severe ischemic MPI increased with increasing CAC of 0, 1-400, and ≥400, respectively (*lower left*). TAC increased with increasing severity of ischemic MPI (*upper middle*); and the proportions of both mild-to-severe ischemic MPI and moderate-to-severe ischemic MPI increased with increasing TAC of 0, 1-1000, and ≥1000, respectively (*lower middle*). On the other hand, CFR significantly decreased with increasing severity of ischemic MPI (*upper right*); and the proportions of both mild-to-severe ischemic MPI and moderate-to-severe ischemic MPI increased with decreasing CFR of ≥2.2, 2.2-1.8, and <1.8, respectively (*lower right*)

**Figure 2:**
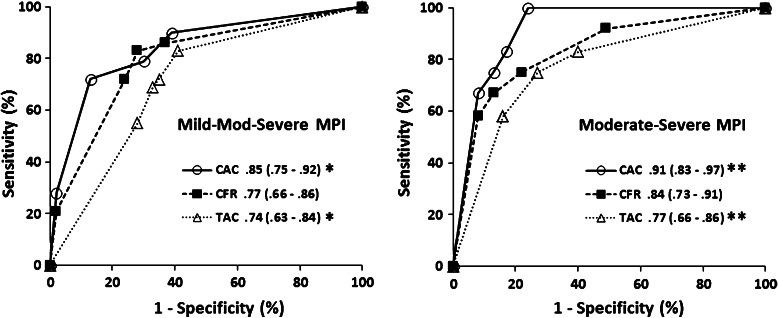
ROC curves for detection of total mild-to-severe ischemic MPI (*left*) and moderate-to-severe ischemic MPI (*right*) by CAC, TAC, and CFR. Area under curve is 0.85 for CAC, and 0.77 for TAC (*P = .05) for mild-to-moderate ischemic MPI; and 0.91 for CAC and 0.77 for TAC for moderate-to-severe ischemic MPI (**P = .04). Values in *parenthesis* represent the range for 95% confidence interval

With increasing severity of ischemic MPI, CAC, and TAC were significantly increased (*P* < .0005 and *P* = .003, respectively), while CFR was decreased (*P* < .0005) (Figure 1A). The proportion of both mild-to-severe ischemic and moderate-to-severe ischemic MPI was increased with increasing CAC and TAC, respectively, but with decreasing CFR (Figure 1B). The sensitivity and specificity of 79%, and 70% of CAC for ischemic MPI with optimal cut-off CAC values of 20 was comparable, respectively, with 83% and 72% of CFR as cut-off of 1.97 and significantly higher than 72% and 65% of TAC with cut-off TAC of 100 (*P* = .05) (Figure 2A; Table 2). The ROC curve analysis showed that the CAC has significantly higher diagnostic accuracy than TAC for the detection of total mild-to-severe ischemic and moderate-to-severe ischemic MPI (*P* = .05 and *P* = .04, respectively). According to the optimal cut-off point of the ROC curve, the sensitivity and specificity of each CAC, CFR, and TAC to predict total mild-to-severe as well as moderate-to-severe ischemic MPI showed higher negative predictive values than positive predictive values (Table 2).

**Table 2:** ROC curve analysis of diagnostic performance for detection of total ischemic MPI and moderate-to-severe ischemic MPI

	Mild, moderate & severe			Moderate & severe		
	CAC	TAC	CFR	CAC	TAC	CFR
Sensitivity (%)	79 (60–92)	72 (53–87)	83 (64–94)	83 (52–97)	75 (43–94)	83 (52–97)
Specificity (%)	70 (54–82)	65 (50–79)	72 (57–84)	83 (71–91)	73 (60–83)	62 (49–74)
PPV (%)	62	57	65	48	35	29
NPV (%)	84	79	87	96	94	95
AUC	.85 (.75–.92)*	.74 (.63–.84)*	.77 (.66–.86)	.91 (.83–.97)**	.77 (.66–.86)**	.84 (.73–.91)
Cut-Off	20	100	1.97	100	250	1.94

Values in parenthesis are 95% confidence interval of the parameters.

*PPV*, Positive predictive value; *NPV*, negative predictive value; *AUC*, area under the curve.

* *P* = .05 and ** *P* = .04 for comparison of CAC and TAC in ischemic MPI and moderate-to-severe ischemic MPI, respectively.

**Figure 3:**
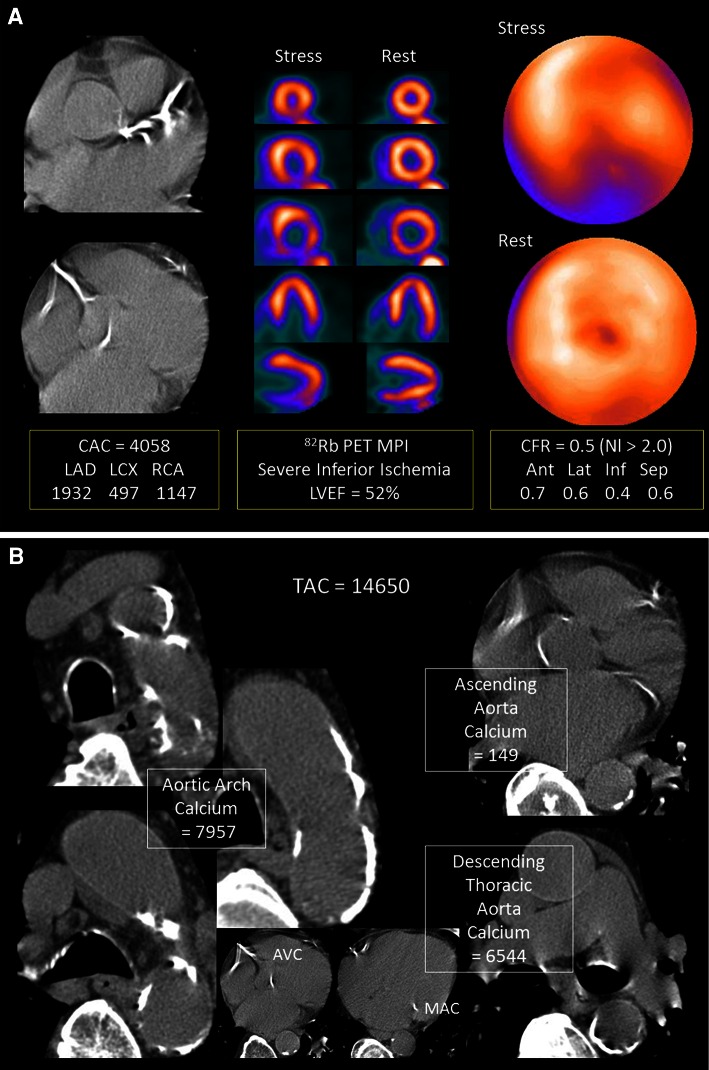
Severe extensive CAC in all three coronary vessels (**A**, *left*) as well as TAC in the ascending aorta, aortic arch, and the descending thoracic aorta including aortic valve (AVC) and mitral annulus calcium (MAC) (**B**) were noted in a 79-year-old male patient with multiple RF of diabetes, hypertension, and hyperlipidemia. Myocardial perfusion images showed evidence of moderate-to-severe ischemia in the inferior wall with transient ischemic dilatation of LV cavity suggesting more extensive disease (**A**, *middle*). The CFR was measured as less than 1.0 in all three coronary territories, indicating coronary steal phenomenon (**A**, *right*). Coronary catheterization and angiography showed evidence of subtotal occlusion of the RCA, extensive disease (90% stenosis) in the LAD territory, as well as evidence of significant disease (70% stenosis) in the LCX territory

A representative case showed severe extensive CAC in all three coronary vessels (Figure 3A, left) as well as TAC in the ascending aorta, aortic arch, and the descending thoracic aorta including aortic valve and mitral annulus calcium (Figure 3B) in a 79-year-old male patient with multiple RF of diabetes, hypertension, and hyperlipidemia. Myocardial perfusion images showed evidence of moderate-to-severe ischemia in the inferior wall with mild transient ischemic dilatation of LV cavity suggesting more extensive disease (Figure 3A, middle). The CFR was measured as less than 1.0 in all three coronary territories (Figure 3A, right). Coronary catheterization and angiography showed evidence of subtotal occlusion of the RCA, extensive disease (90% stenosis) in the LAD territory, as well as evidence of significant disease (70% stenosis) in the LCX territory.

### Intercorrelation Between CAC, TAC, and CFR

**Figure 4:**
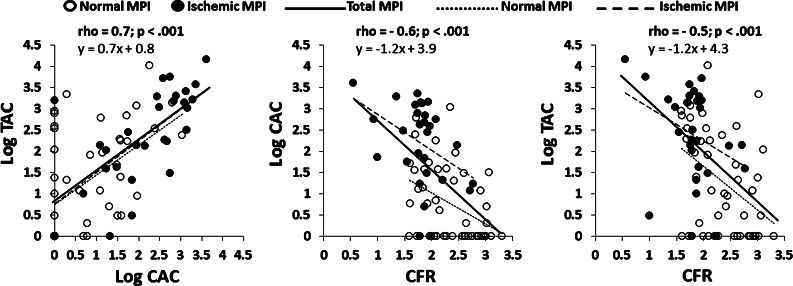
Significant correlations existing between global CAC and global TAC in all 75 patients, as well as the 46 patients with normal MPI and the 29 patients with abnormal MPI (*left*). Significant negative correlations existing between global CFR and CAC in all 75 patients, as well as the 46 patients with normal MPI and 29 patients with abnormal MPI (*middle*). Significant negative correlations of global CFR with global TAC in all 75 patients, as well as patients with normal MPI studies and patients with abnormal MPI scans (*right*)

There were significant correlations between global CAC and TAC (Figure 4A) in all 75 patients (ρ = 0.7; *P* < .001), as well as in patients with normal (ρ = 0.5; *P* < .001) and ischemic (ρ = 0.7; *P* < .001) MPI. There were also significant correlations between global CAC and regional TAC (ρ = 0.6; *P* < .001), including ascending aorta, aortic arch, and descending thoracic aorta (ρ = 0.6, respectively; *P* < .001 in each region). There was a significant inverse correlation between global CFR and CAC (Figure 4B) in all 75 patients (ρ = −0.6; *P* < .001), as well as in patients with normal MPI (ρ = −0.4; *P* = .02) and those with ischemia (ρ = −0.4, *P* = .003). There was a significant negative correlation between regional CFR and CAC of the corresponding coronary arteries in all 75 patients (total of 225 coronary vessel territories) (ρ = −0.5; *P* < .001) regarding LAD, LCX, and RCA territories (ρ = −0.5, respectively; *P* < .001 in each coronary territory), as well as in patients with normal MPI (138 vessel territories) (ρ = −0.3; *P* < .001) and those with ischemia (87 vessel territories) (ρ = −0.4; *P* < .001). There was also a significant negative correlation of global CFR with global TAC (Figure 4C) in all 75 patients (ρ = −0.5, *P* < .001), as well as in patients with normal studies (ρ = −0.4; *P* = .008) and in ischemic patients (ρ = −0.4; *P* = .05), as well as with regional TAC (ρ = −0.5; *P* < .001), including ascending aorta (ρ = −0.4; *P* < .001), aortic arch (ρ = −0.4; *P* < .001) and descending thoracic aorta (ρ = −0.5; *P* < .001).

### CAC, TAC, and CFR by Age and Gender: Predictors of Ischemia

**Figure 5:**
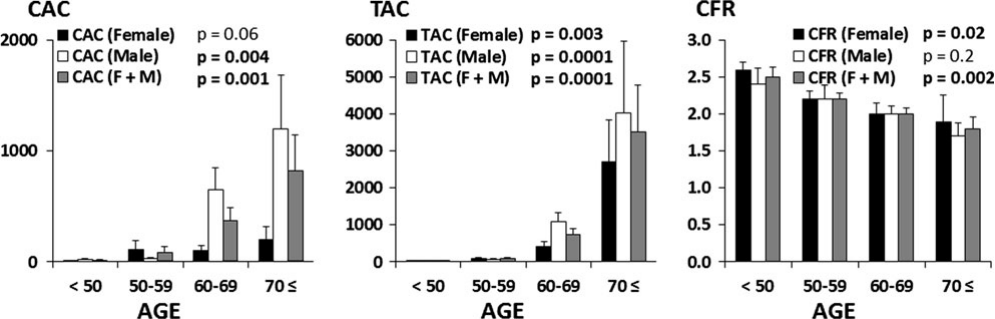
Both the mean CAC and TAC scores significantly increased, (*left* and *middle*, respectively), while the mean CFR decreased (*right*) with increasing age. By gender-specific analysis, the increasing CAC with age was significant in male patients, but did not reach statistical significance in female patients (*left*). The increasing TAC with age was significant both in female and male patients, respectively (*middle*). On the other hand, the decrease of CFR was significant in females, but did not reach the statistical significance in male patients (*right*). *F*, Female, *M*, male

Global CAC and TAC scores increased significantly (*P* = .001 and *P* = .0001, respectively) (left and middle), while CFR decreased (*P* = .002) (right) with increasing age (Figure 5A). By gender-specific analysis, age-related increase of CAC was found significant in males, but did not reach statistical significance in female patients (*P* = .06). Age-related increase of TAC was significant in both female and male patients (*P* = .003 and *P* = .0001, respectively) (Figure 5B). On the other hand, the decrease of CFR observed with age was significant in females (*P* = .002), but did not reach statistical significance in male patients (*P* = .2) (Figure 5C).

By gender-specific univariate regression analysis, age (*P* = .001), CAC (*P* = .004) and CFR (*P* = .008) in males, and CFR (*P* = .0001), age (*P* = .002) and TAC (*P* = .01) in females were significant predictors for abnormal MPI (Table 3). By backward stepwise multiple logistic regression analysis, the most potent predictor of abnormal PET-MPI was CFR [odds ratio (OR) = 0.17, *P* = .01], followed by age (OR = 1.07, *P* = .02), gender (OR = 4.01, *P* = .03) and CAC (OR = 1.002, *P* = .9).

**Table 3:** Univariate predictors of ischemia

	OR	*P* value	CI
CFR (≤2)	0.13	.0001	0.04–0.36
Age (≥65)	9.00	.0001	3.06–26.59
CAC (>50)	12.47	.0001	4.09–38.04
Gender (male)	3.79	.006	1.41–10.18
TAC			
Quartile 2 vs 1	3.43	.17	0.59–19.80
Quartile 3 vs 1	5.82	.046	1.03–32.79
Quartile 4 vs 1	20.80	.001	3.45–125.29
Diabetes mellitus	1.67	.3	0.60–4.66
Hypertension	1.56	.4	0.59–4.17
Hyperlipidemia	1.50	.4	0.58–3.87
Smoking	1.21	.8	0.25–5.85
Family history	2.20	.2	0.63–7.65
BMI (≥25)	1.01	1.0	0.34–3.00
LVEF (<40)	0.60	.2	0.11–3.22

* For abbreviations, see Table 1.

## Discussion

The present study demonstrated that the degree of ischemia on PET MPI is directly proportional to the global CAC and TAC and inversely related to CFR. There was significant correlation between CAC and TAC, and both of them were inversely associated with CFR. Furthermore, global CFR was the strongest predictor for ischemic MPI followed by CAC in males and TAC in females, respectively. Therefore, combination of MPI, CFR, and CAC as well as TAC measurements may have a complementary role in the management of patients with intermediate risk for CAD. In fact, the time course of CAD progression or regression based on changes of calcification, soft-plaque formation, and endothelial function could be non-invasively monitored as comprehensive end-points in the evaluation of new and established therapies.

For CAC to detect ≥50% stenosis on coronary angiography, the previously reported AUC from ROC curve analysis using MDCT and EBCT were .85 and .83, respectively, with the same sensitivity of 80% and specificity of 86% (optimal cut-offs of 198 and 291, respectively).^21^ These results are in line with the present study as the AUC and sensitivity and specificity of CAC were .85, 79%, and 70% for total ischemic MPI, (Figure 3) and 0.91, 83%, and 83% for moderate-to-severe ischemic MPI, with optimal cut-off CAC values of 20 and 100, respectively (Figure 2; Table 2). In asymptomatic patients without obstructive CAD, CAC and MPI were independent and complementary predictors of short- and long-term cardiac events,^22^ but there is a previous article showing a lack of correlation.^23^ A significant correlation reported between TAC and CAC^12^,^14^,^24^,^25^ reproduced in the current study supports a link between aortic and coronary plaque instability^11^ related with CVD.^13^ However, the failure of TAC to further improve event prediction over CAC^14^ could be explained by the significantly higher diagnostic accuracy of CAC than TAC for detection of ischemic MPI in the current study (Figure 2).

Curillova et al,^26^ reported a significant but weak inverse correlation coefficients between CAC and CFR in normal ^82^Rb PET MPI (*r* = −0.3, *P* ≤ .001 in global; and *r* = −0.2, *P* ≤ .001 in regional measurements), similar to the current data in normal MPI (ρ = −0.4; *P* = .02 in global, and ρ = −0.3; *P* < .001 in regional measurements) (Figure 3B). In the patients without obstructive CAD, the inverse association between CFR and CAC disappeared after adjusting for age, gender, body mass index (BMI), and conventional RF in symptomatic individuals,^27^ and also weakened in patients with an intermediate risk of CAD.^26^ In asymptomatic subjects, the association was attenuated with advancing age,^28^ and also related only to age but not to CAC in those with a family history of CAD,^29^ suggesting that the association is largely due to overlapping clinical RF, although each of these measurements carry largely independent information. In the current gender-specific univariate analysis, the age, CAC, and CFR in males but the CFR, age, and TAC in females were significant predictors for ischemic PET MPI, because of a higher prevalence and magnitude of TAC compared to lower CAC in pre- and peri-menopausal women (Figure 5). Finally, multiple logistic regression revealed the most potent predictor is CFR, followed by age, gender, and CAC. This invaluable role of CFR could be referred to the FAME trial which successfully demonstrated that a functional flow-guided intervention leads to better outcomes than the one based on anatomic severity of stenosis^30^ as well as a reported “warranty” period of 3 years of normal CFR for a long-term prognostic cardiovascular outcome.^31^

**Figure 6:**
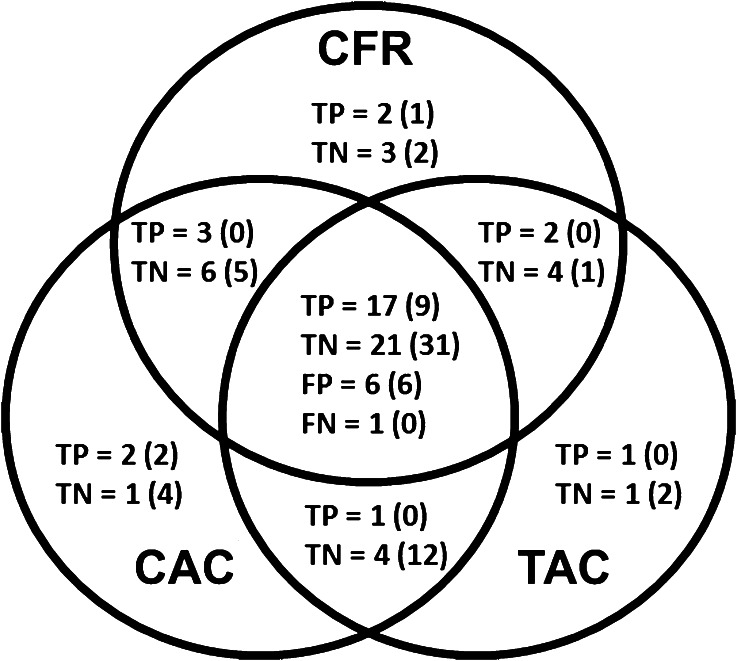
Diagrams of CFR, CAC and TAC for detection of total mild-moderate-severe ischemic MPI in 75 patients. Those for detection of moderate-to-severe ischemic MPI were expressed in the *parentheses*. The triple measurements were correct and matched in 51% (53%) of the patients, and only single measurement is correct in 13% (15%). *TP*, True positive, *TN*, true negative, *FP*, false positive, *FN*, false negative

For detection of total ischemic MPI, and only moderate-to-severe ischemic MPI, the triple measurements of CFR, CAC, and TAC were correct and matched in 51% and 53%, respectively, whereas only a single measurement was correct in 13% and 15%, which appears complementary (Figure 6), though the outcome analysis is needed to be validated through a study in a larger population. This relationship between vasoreactivity of CFR and vascular calcification of CAC/TAC may reflect biologically different temporal and spatial processes of atherosclerosis with aging in a gender-specific manner.^27^,^28^

### Study Limitations

First, our study of cardiac PET/CT consisted of physician referral-based patient population with an intermediate likelihood of CAD, and hence, it comes with selection bias to limit generalizability of our results to screen low risk population. Second, an absolute rest or stress myocardial blood flow was not investigated in depth using our in-house software, given highly variable values at rest and during stress depending on each software due to its own methodology compared to highly consistent CFR values.^32^ Finally, it must be emphasized that the studied patient population was too small to draw definite conclusions; rather, our data consolidated the prior reports of the relationship between myocardial perfusion, vasoreactivity, and vascular calcifications.

## Conclusion

The risk of ischemia by MPI increases with increasing CAC and TAC. However, the main contributing factor of ischemia is reduced CFR. On the other hand, not only due to the blunted and delayed increase of CAC than TAC with age in women, but also the blunted decrease of CFR with age in men, the CAC in males, and the CFR in females are supposed to the best predictors for detection of abnormal PET-MPI, respectively. Therefore, the combination of MPI, CFR, and CAC as well as TAC from a single PET/CT scanning may have a complementary role in the management of patients with intermediate risk of CAD.

## Electronic supplementary material

Below is the link to the electronic supplementary material.

Supplementary material 1 (DOC 33 kb)
