# Supplementary material for: Coronary artery and thoracic aorta calcification is inversely related to coronary flow reserve as measured by 82Rb PET/CT in intermediate risk patients
Source: J Nucl Cardiol. 2013 Mar 7;20(3):375–84. doi: 10.1007/s12350-013-9675-5 (PMC3653061; doi:10.1007/s12350-013-9675-5)
Supplement: Supplementary file 1 — Supplementary material 1 (DOC 33 kb) [file 12350_2013_9675_MOESM1_ESM.doc]

**SUPPLEMENT MATERIALS**

Findings similar to those reported here and in our preliminary results (see below) were recently reported.

Preliminary findings from this study were initially presented as abstracts in 2006 and 2007, cited below:

1. 53rd Annual Meeting, Society for Nuclear Medicine, San Diego, CA June 3-7, 2006

Citation: Kim JH, Machac J, Kim SC, Travis A, Rafique A, Quispe M, Colon D, Krynyckyi BR, Kim CK.

Inverse Relationship between Coronary Flow Reserve and Coronary Artery Calcium according to Severity of Abnormal ^82^Rb PET/CT in Intermediate Risk Patients. J Nucl Med 2006;47(1):205p

2. 56th Annual Meeting, American College of Cardiology, New Orleans, LO, March 24-27, 2007.

Kim JH, Travis A, Rafique A, Machac J.

Citation: Global and Regional Thoracic Aortic as well as Coronary Calcifications, are inversely related with Coronary Flow Reserve measured by Rb-82 PET/CT in Intermediate Risk Patients. JACC 2007;49(9):101A

**METHODS**

Two hundred and sixteen consecutive patients who completed combined rest-stress ^82^Rb PET/CT MPI from March 2005 to July 2006 at Mount Sinai Hospital, NY, were reviewed. All patients were referred for pharmacologic (dipyridamole or adenosine) stress PET/CT MPI on clinical grounds. Global and regional CFR, CAC and TAC scorings were measured. A total of 141 patients were excluded for complicating diagnoses as follows: cardiac transplantation (n=44), end stage renal disease (n=38), end stage liver disease (n=31), previous percutaneous coronary intervention (n=23) and coronary artery bypass graft (n=5). Therefore, 75 patients were included in the final analysis.

**Coronary artery and thoracic aorta calcium scoring**

Scoring was performed by an experienced independent observer who was blinded to the patients’ clinical history, outcomes, and PET scan results. CAC scores were calculated for the left anterior descending (LAD) artery (including calcium present in the left main coronary artery), left circumflex (LCX) artery, and right coronary artery (RCA) and then summed to provide a global/total CAC score for each patient. CAC percentile scores were then assigned based on age and sex.

**Quantification of Coronary Flow Reserve (CFR)**

A comparison of the simple model with the compartmental model yielded a good correlation (r = 0.7; p < 0.001), reproducibility (r = 0.9), and lower susceptibility to noise than the compartmental model (unpublished data). Using a similar method of measuring ^82^Rb retention, ^33^ showed a good reproducibility and good correlation with microsphere flow measurements (r = 0.7; p = 0.001) in animal studies.

**Statistical Analysis**

For multiple logistic regression analysis, models were evaluated for goodness of fit. Also, odds ratios were calculated to explore the risk of having abnormal MPI among all patients as well male and female subgroups, separately. The optimal thresholds were for CAC, ≥ 50 vs. < 50 ^34^; for CFR, < 2.0 vs. ≥ 2.0 ^20^; for age, ≥ 65 vs. < 65; for LVEF, ≥ 40% vs. < 40%; and for BMI, ≥ 25 vs < 25. Since no accepted thresholds or cut-offs exist for TAC risk categories, we chose to divide TAC scores into quartiles to maximize comparability. The lowest quartile of TAC was used as reference categories. Cut-offs for TAC score quartiles were 0–2 (1st quartile), 3–92 (2nd quartile), 93–796 (3rd quartile), and > 796 (4th quartile).

**References**

**33. deKemp RA, Ruddy TD, Hewirtt T, Dalipaj MM, Beanlands RS. Detection of serial changes in absolute myocardial perfusion with ^82^Rb PET. J Nucl Med. 2000;41:1426-35.**

**34. Walker NJ, Sites FD, Shofer FS, Hollander JE. Characteristics and outcomes of young adults who present to the emergency department with chest pain. Acad Emerg Med. 2001;8:703-8.**
